# A Comparative Analysis of Neuroprotective Properties of Taxifolin and Its Water-Soluble Form in Ischemia of Cerebral Cortical Cells of the Mouse

**DOI:** 10.3390/ijms241411436

**Published:** 2023-07-14

**Authors:** Elena G. Varlamova, Nina I. Uspalenko, Natalia V. Khmil, Maria I. Shigaeva, Mikhail R. Stepanov, Mikhail A. Ananyan, Maria A. Timchenko, Maxim V. Molchanov, Galina D. Mironova, Egor A. Turovsky

**Affiliations:** 1 Institute of Cell Biophysics, Russian Academy of Sciences, Pushchino 142290, Russia; 1928lv@mail.ru; 2 Institute of Theoretical and Experimental Biophysics, Russian Academy of Sciences, Pushchino 142290, Russia; nina_uspalenko@mail.ru (N.I.U.); nat-niig@yandex.ru (N.V.K.); shigaeva-marija@rambler.ru (M.I.S.); maria_timchenko@mail.ru (M.A.T.); lvlaks.m@gmail.com (M.V.M.); 3 Advanced Technologies Ltd., Moscow 119333, Russia; nanotech@nanotech.ru (M.R.S.); nanoindustry@mail.ru (M.A.A.)

**Keywords:** taxifolin, aqua taxifolin, oxygen–glucose deprivation, reoxygenation, gene expression, cortex, astrocytes, neurons, calcium, necrosis, apoptosis

## Abstract

Cerebral ischemia, and, as a result, insult, attacks up to 15 million people yearly in the world. In this connection, the development of effective preventive programs and methods of therapy has become one of the most urgent problems in modern angiology and pharmacology. The cytoprotective action of taxifolin (TAX) in ischemia is well known, but its limitations are also known due to its poor solubility and low capacity to pass through the hematoencephalic barrier. Molecular mechanisms underlying the protective effect of TAX in complex systems such as the brain remain poorly understood. It is known that the main cell types of the brain are neurons, astrocytes, and microglia, which regulate the activity of each other through neuroglial interactions. In this work, a comparative study of cytoprotective mechanisms of the effect of TAX and its new water-soluble form aqua taxifolin (aqTAX) was performed on cultured brain cells under ischemia-like conditions (oxygen–glucose deprivation (OGD)) followed by the reoxygenation of the culture medium. The concentration dependences of the protective effects of both taxifolin forms were determined using fluorescence microscopy, PCR analysis, and vitality tests. It was found that TAX began to effectively inhibit necrosis and the late stages of apoptosis in the concentration range of 30–100 µg/mL, with aqTAX in the range of 10–30 µg/mL. At the level of gene expression, aqTAX affected a larger number of genes than TAX; enhanced the basic and OGD/R-induced expression of genes encoding ROS-scavenging proteins with a higher efficiency, as well as anti-inflammatory and antiapoptotic proteins; and lowered the level of excitatory glutamate receptors. As a result, aqTAX significantly inhibited the OGD-induced increase in the Ca^2+^ levels in the cytosol ([Ca^2+^]_i_) in neurons and astrocytes under ischemic conditions. After a 40 min preincubation of cells with aqTAX under hypoxic conditions, these Ca^2+^ signals were completely inhibited, resulting in an almost complete suppression of necrotic death of cerebral cortical cells, which was not observed with the use of classical TAX.

## 1. Introduction

It is known that 85% of strokes are caused by the occlusion of a cerebral artery. A rapid restoration of blood supply is currently the treatment of choice to reduce the area of a brain’s damage after a stroke. Nevertheless, two thirds of stroke survivors remain essentially disabled with a low quality of life [1]. However, although the timely restoration of blood flow in the cerebral artery is necessary for the recovery of an ischemic tissue, the reperfusion of the ischemic region may also aggravate tissue injury with a further worsening of disease outcome [2]. Thus, on the one hand, reperfusion increases the content of oxygen and glucose in the tissue; on the other hand, it also enhances the inflammatory response. This problem arises after the spontaneous or intervention-induced lysis of a thrombus and after successful mechanical thrombectomy. However, the basic cellular and molecular processes and their relationships with neuronal survival are still poorly understood.

It is known from the literature that inflammation can be associated with the development of oxidative stress and that antioxidants can significantly cup off this process [3]. Among these is dihydroquercetin (3,5,7,3′,4′-pentahydroxyflavanone (DHQ), also known as taxifolin (TAX)), which has powerful antioxidant, organ-protective, and anti-inflammatory effects [3]. Its application in the form of biologically active additives showed a high efficacy as cerebral protective agents [4]. Antioxidants belong to the class of polyphenols, which are abundant in olive oil, grapes, citrus fruits, and onions, as well as in Siberian larch, *Larix sibirica*, *Pinus roxburghii*, *Cedrus deodara* Chinese yew, etc. [5,6].

The antioxidant taxifolin (TAX) has found wide use in clinical practice due to a great number of positive pharmacological properties, among which, in addition to its antioxidant effect, are the anticancer action and the capacity to inhibit angiogenesis and apoptosis and regulate the cell cycle [7,8]. At the level of neuronal networks, TAX produces a complex neuroprotective effect through a combination of several mechanisms: a rapid suppression of reactive oxygen species (ROS) production due to its high antioxidant properties and the inhibition of irreversible increases in the Ca^2+^ levels in the cytosol of GABAergic neurons, which prevents their death and subsequent hyperexcitation of the entire network [9]. It is believed that the protective effect of this antioxidant is accomplished through changes in the expression of anti-apoptotic genes, the posttranslational modification of the subunits of AMPA receptors, and the inhibition of apoptosis in populations of GABAergic neurons, which are most vulnerable to ischemia [9].

With all these positive properties, classical TAX shows weak bioaccessibility when administered both intravenously and, particularly, perorally. After the intravenous administration of TAX to rats, its maximum concentrations in the plasma, kidneys, liver, heart, brain, and skeletal muscles are reached as late as 24 h after the injection [10,11]. Therefore, for activating the positive effects, TAX is used in clinical practice at high concentrations (hundreds of micromoles). This may be related to its poor permeation through the blood–brain barrier [12].

In view of this, much attention is recently being given to the search for effective methods of delivering TAX to the brain. Among these are liposomal delivery, the production of nanocapsules, and the doping of nanoparticles with TAX [13]. In addition, water-soluble forms of TAX were created, in which solubility was increased due to structural changes [14,15,16]. It was assumed that the production of water-soluble forms of TAX would be one of the least expensive and more effective methods of its delivery to brain cells. Indeed, it was shown that increasing the solubility of TAX due to changes in its structure restored the activity of mitochondrial enzymes in old rats to the level observed in young animals [16].

We have shown recently that a water-soluble complex of TAX with polyvinylpyrrolidone (PVP) aqTAX produces a marked antioxidative effect, preventing the development of lipid peroxidation in the blood serum in experimental cardiomyopathy [17]. The goal of the present work was a comparative study of the molecular mechanisms of the cytoprotective effect of aqTAX and of the classical (powder) form of TAX in a model of ischemia and reoxygenation in mouse brain cell cultures. Preliminarily, the state of TAX in aqTAX was determined by NMR spectroscopy.

## 2. Results

### 2.1. Effectiveness of the Cytoprotective Action of 24 h Incubation of Cells with Different Concentrations of TAX and aqTAX on the Survival of Cerebral Cortical Cells after 2 h OGD and 24 h Reoxygenation

**Figure 1:**
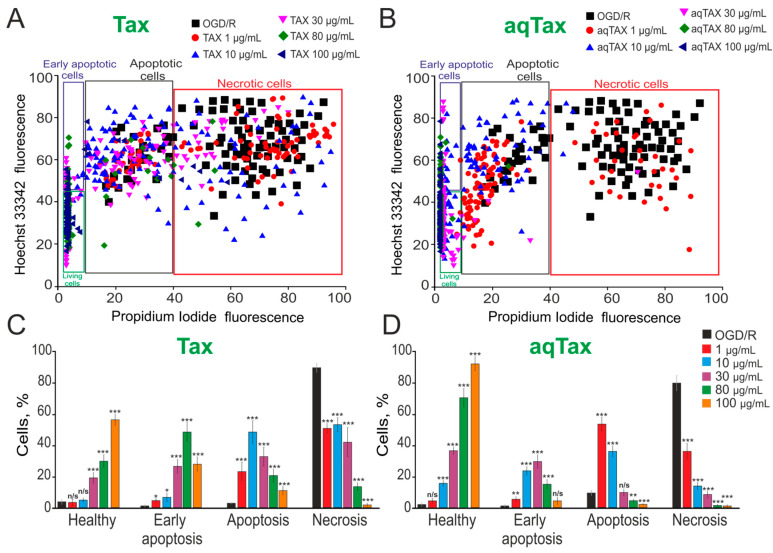
Cytoprotective action of 24 h incubation of cells at different concentrations of TAX and aqTAX on the survival of cerebral cortical cells after 2 h OGD and 24 h reoxygenation (OGD/R). (**A**,**B**)—Cytograms demonstrating the viability of cultured cortical primary cells after OGD/R and OGD/R depending on preincubation with different concentrations of TAX (**A**) and aqTAX (**B**). *X*-axis—the intensity of PI fluorescence; *Y*-axis—the intensity of Hoechst 33342 fluorescence. Cells were stained with probes 24 h after OGD/R. (**C**,**D**)—Effect of TAX (**C**) and aqTAX (**B**) on the induction of necrosis and apoptosis 24 h after OGD/R. The number of cell cultures 5 (N); the number of coverslips with cells for each sample 5 (n). For panels (**C**,**D**), the results are presented as mean ± SEM. Cell images are presented in Supplementary Materials (Figures S1 and S2). Statistical significance was assessed using *t*-test. Black asterisks indicate the differences between the experimental groups compared with the OGD/R group. *** *p* < 0.001, ** *p* < 0.01, and * *p* < 0.05. n/s—insignificant differences.

It was shown that incubation under ischemia-like conditions (oxygen–glucose deprivation, OGD) for 2 h followed by 24 h reoxygenation (OGD/R) led to the necrotic death of about 90% of cerebral cortical cells, with the remaining cells being at early and late stages of apoptosis (Figure 1A,C and Figure S1). A preliminary (for 24 h) incubation of these cells with TAX at concentrations of 1 and 10 µg/mL decreased the necrotic death of cells from 90% to 43–48%; however, their great portion was at the late stage of apoptosis, and the number of viable cells did not increase (Figure 1A,C and Figure S1). Increasing the concentration of TAX to 30 µg/mL improved the survival of cerebral cortical cells by 20%. In this case, the number of cells at the early and late stages of apoptosis amounted to 24% and 32%, respectively, whereas necrosis was registered in 37% of cells (Figure 1A,C and Figure S1). A preincubation of cortical cells under hypoxic conditions with TAX (80 µg/mL) increased the number of viable cells to 35%. Early and late apoptosis were recorded in 38% and 18% of cells and necrosis only in 12%. A preincubation with TAX at a concentration of 100 µg/mL produced the most pronounced effect on survival, when the amount of viable cells amounted to about 60%. Necrosis under these conditions was almost completely suppressed, and apoptosis was recorded at the early and late stages only in 27% and 12% of cells, respectively (Figure 1A,C and Figure S1).

A preincubation of cortical cells with the water-soluble form of TAX (aqTAX) at a concentration of 1 µg/mL did not significantly affect cell survival after OGD/R. However, even at this concentration, necrotic cell death decreased to 30%; 8% and 52% of cells were at the early and late stages of apoptosis, respectively (Figure 1B,D and Figure S2). Increasing the aqTAX concentration to 10 and 30 µg/mL decreased necrotic cell death to 12% and 9%, respectively. Early and late apoptosis, in this case, was recorded in 21% and 34% of cells at a concentration of 10 µg/mL and in 30% and 14% of cells at a concentration of 30 µg/mL (Figure 1B,D and Figure S2). At concentrations of 80 and 100 µg/mL, aqTAX almost completely suppressed necrotic cell death and late stages of apoptosis, and only 17% and 7% of cells were at early reversible stages of apoptosis, respectively (Figure 1B,D and Figure S2).

Thus, it was evident that aqTAX had much more pronounced antinecrotic and antiapoptotic effects compared with water-insoluble TAX, and these effects were recorded at concentrations as low as 10 µg/mL.

### 2.2. A Comparative PCR Analysis of the Effect of TAX and aqTAX on the Expression of Genes Encoding the Proteins of the Redox Status, Apoptosis, Necrosis, Inflammation, and the Subunits of Receptors in Cerebral Cortical Cell Cultures in Normoxia

**Figure 2:**
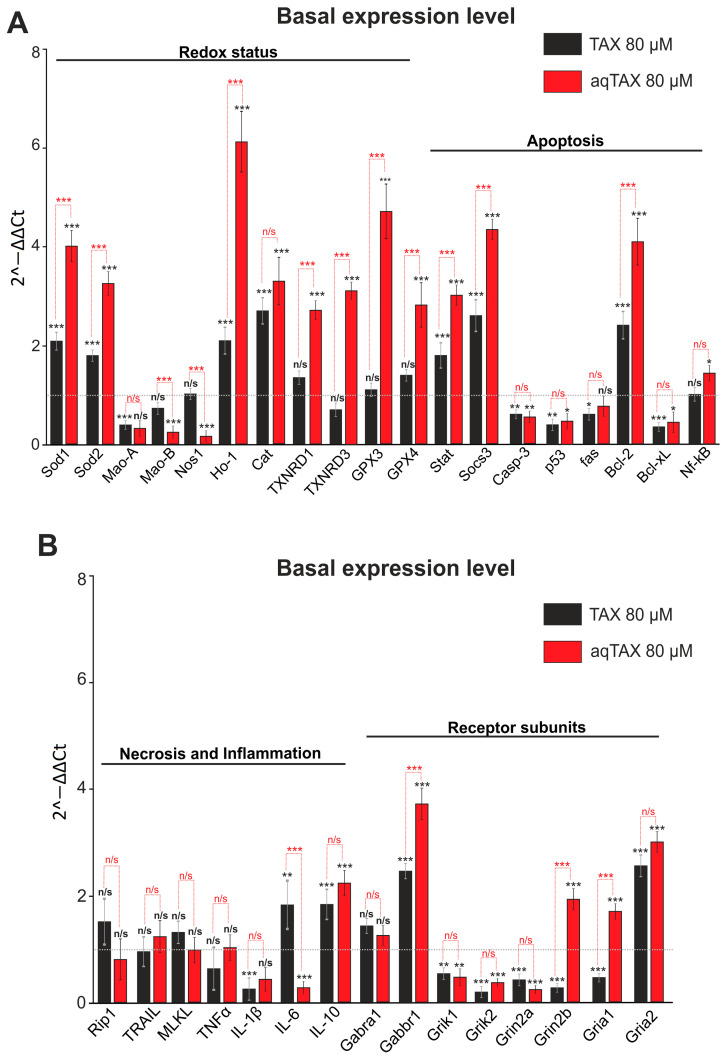
Effect of TAX (80 µM) and its water-soluble form aqTAX (80 µM) on the basic level of expression of genes encoding the proteins of redox status, apoptosis (**A**), necrosis, inflammation, and receptor subunits (**B**) in cultured cortical cells. Cortical cells were incubated for 24 h with TAX and aqTAX. The expression level in the control (cells were not exposed to the compounds) is taken to be unity (a grey dotted line). The number of samples (single cell cultures) is three. Statistical significance was assessed using *t*-test. Black asterisks indicate the differences between the experimental groups compared with the Control group. Differences between experimental groups are marked with red asterisks. *** *p* < 0.001, ** *p* < 0.01, and * *p* < 0.05. n/s—insignificant differences.

In accordance with the dose–effect dependences for the two TAX forms, recorded in the study, a dose of 80 µg/mL was used in the PCR analysis for both compounds. After a 24 h exposure of cells to TAX and aqTAX, total RNA was isolated from cell cultures. It was found that the administration of TAX into culture medium under normoxia conditions increased the basic level of the expression of genes encoding the antioxidant proteins Sod1, Sod2, Ho-1 (hemoxygenase), and Cat (catalase) by 2.1, 1.8, 2.1, and 2.7 times, respectively, compared with the control (Figure 2A, black bars). aqTAX at a similar concentration affected the basic level of expression of the genes of the redox status much more significantly, so the expression of Sod1, Sod2, Ho-1, and Cat increased by 4, 3.2, 6.1, and 3.3 times, respectively, compared with the control (Figure 2A, red bars). The levels of the expressions of the genes encoding thioredoxin reductases 1 and 3 (TXNRD1 and TXNRD3), as well as glutathione peroxidases 3 and 4 (GPX3 and GPX4) in the presence of TAX, remained almost unchanged, whereas aqTAX increased this characteristic by 2.7, 3.1, 4.7, and 2.8 times, respectively (Figure 2A). The expression of genes of proteins having prooxidant properties (Mao, Nos1) decreased, but in the presence of aqTAX more significantly than by the action of TAX (Figure 2A).

The basic levels of the expressions of the genes encoding proapoptotic proteins (Casp-3, p53, fas, Bcl-xL) decreased by the actions of both TAX and aqTAX, and the levels of expressions of the genes of the antiapoptotic proteins (Stat, Socs3, Bcl-2) after incubation with aqTAX increased more strongly (Figure 2B). The basic levels of expressions of the genes encoding the marker proteins of necrosis and inflammation either did not change or decreased slightly after the incubation of cortical cells with both forms of TAX. The expressions of the genes encoding the subunits of the excitatory glutamate receptors NMDA, AMPA, and KAR decreased more markedly by the action of aqTAX (Figure 2B, red bars).

### 2.3. A Comparative PCR Analysis of the Effect of TAX and aqTAX on the Expression of Genes Encoding the Proteins of the Redox Status, Apoptosis, Necrosis, Inflammation, and the Subunits of Receptors in Cerebral Cortical Cell Cultures in Ischemia/Reoxygenation

**Figure 3:**
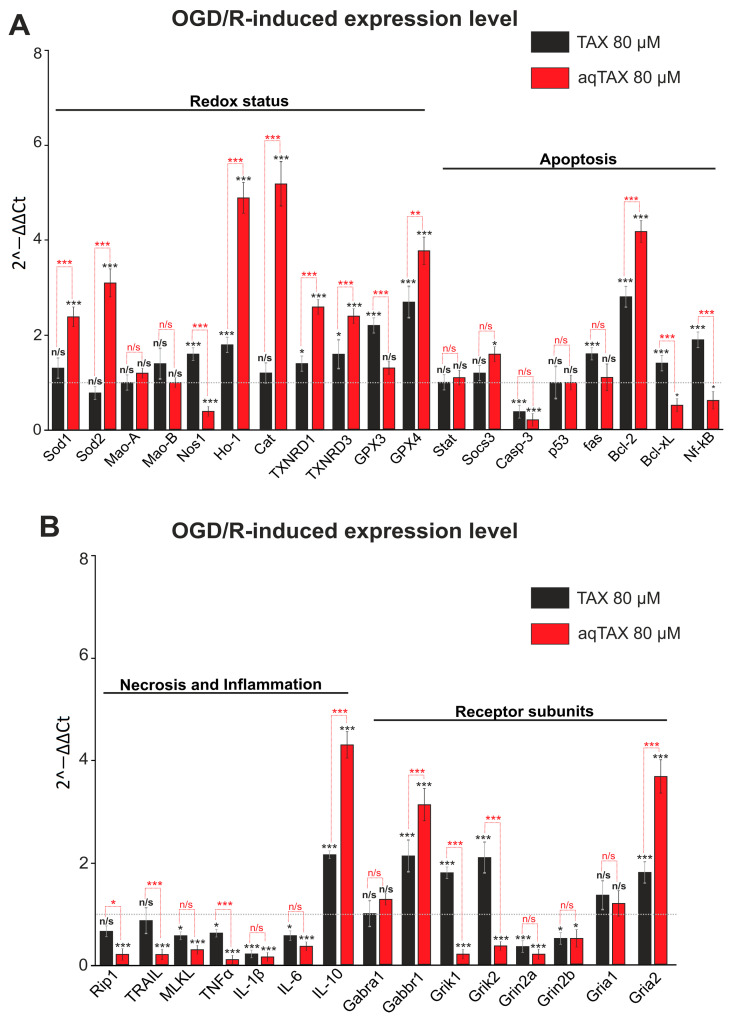
The effect of TAX (80 µM) and its water-soluble form aqTAX (80 µM) on the level of the OGD/R-induced expression of genes encoding the proteins of the redox status, apoptosis (**A**), necrosis, inflammation, and receptor subunits (**B**) in cerebral cortical cells. Cells were incubated for 24 h with TAX or aqTAX; then, OGD conditions were created for 2 h, after which reoxygenation was carried out by transferring the cells into a CO_2_ incubator for 24 h. Then, total RNA was isolated. The expression level in cortical cells after OGD/R without preliminary incubation with the antioxidants (a grey dotted line) was taken to be unity. The number of samples (single cell cultures) was three. Statistical significance was assessed using *t*-test. Black asterisks indicate the differences between the experimental groups compared with the OGD/R group. Differences between experimental groups are marked with red asterisks. *** *p* < 0.001, ** *p* < 0.01, and * *p* < 0.05. n/s—insignificant differences.

To reveal the cytoprotective actions of the two taxifolin forms under OGD/R, experiments were carried out by the following scheme: cells were incubated for 24 h with TAX and aqTAX, after which OGD was induced for 40 min; reoxygenation was carried out by returning the cells into the CO_2_ incubator. Then, the total RNA was isolated from cells, and PCR analysis of gene expression was carried out. As seen from Figure 3A, aqTAX (red bars) after OGD/R induced a more pronounced increase in the expression of genes encoding the antioxidant proteins, as compared with TAX. A similar effect was recorded in the case of the gene for the antiapoptotic protein Bcl-2, and the expression of genes for proapoptotic proteins (Bcl-xL and NF-kB) was reduced only by the action of aqTAX (Figure 3A, red bars).

The most clearly pronounced difference between TAX and aqTAX was observed in the increase in the level of the OGD/R-induced expression of genetic markers of necrosis and inflammation (Figure 3B). As for genes encoding the receptor subunits, the incubation of cells with aqTAX after OGD/R led to a decrease in the expression of the *Grik* and *Grin* genes, whereas the effect of the classical TAX was significantly less pronounced. The level of expression of the *Gabbr1* gene, which encodes the GABA(B) receptor, increased after OGD/R in the presence of both TAX and aqTAX, with the effect of aqTAX being more pronounced (Figure 3B).

Thus, aqTAX induces a significantly more pronounced, compared with TAX, cytoprotective effect on cerebral cortical cells by changing the basic expression and the OGD/R-induced expression of genes encoding antioxidant, antiapoptotic, and anti-inflammatory proteins as well as the subunits of excitatory glutamate receptors.

### 2.4. Comparison of the Effects of Classical TAX and aqTAX on the Ca^2+^ Dynamics in Cerebral Cortex Cells under Ischemia-like Conditions

**Figure 4:**
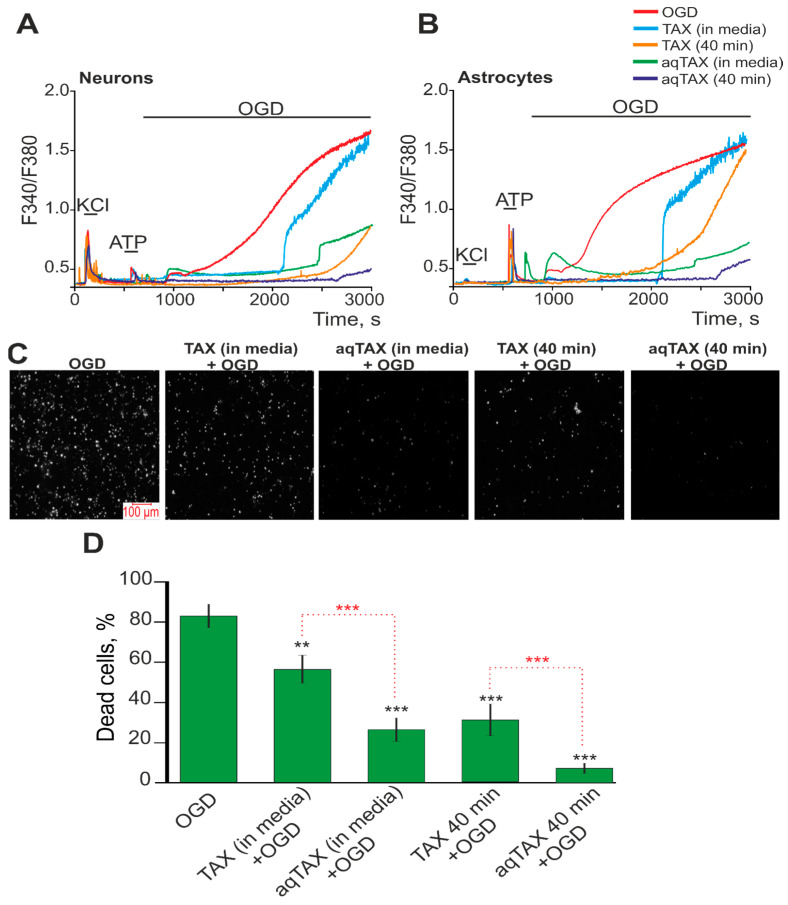
Effect of preliminary incubation of cerebral cortical cells with TAX and aqTAX on the generation of Ca^2+^ signals and survival under OGD conditions. (**A**,**B**)—Ca^2+^ signals of neurons (**A**) and astrocytes (**B**) upon OGD (40 min) and OGD after 40 min of preincubation with 80 µM TAX and 80 µM aqTAX. Short-term applications of 35 mM KCl and 10 µM ATP were used to detect neurons and astrocytes, respectively. Ca^2+^ signals from neurons and astrocytes averaged over several tens of cells are shown. The experiments were performed in triplicates on three separate cell cultures. (**C**)—Images of cortical cell culture in the propidium iodide (PI) fluorescence detection channel in the experimental groups upon 40 min OGD (del treatment) in experimental groups after a 40 min incubation with TAX or aqTAX. White dots represent the PI-stained nuclei of necrotic cells. (**D**)—Effect of a 40 min incubation with TAX and aqTAX on cell viability under hypoxic conditions after 40 min OGD. Statistical significance was assessed using the paired *t*-test. Differences between the experimental groups and the OGD group are shown by black asterisks. Differences between experimental groups are indicated by red asterisks. *** *p* < 0.001, ** *p* < 0.01.

The dynamics of cytosolic calcium ([Ca^2+^]_i_) determine most intracellular processes and are responsible for the activation of signaling pathways of cell survival and cell death [18,19]. The known global increase in [Ca^2+^]_i_ during OGD correlates with the fast necrotic death of neurons and astrocytes [20]. It was shown in the present study that a 40 min OGD led to a biphasic increase in [Ca^2+^]_i_ in neurons (Figure 4A) and astrocytes (Figure 4B). The staining of cells with propidium iodide (PI) after OGD showed more than 80% cell death (Figure 4D) in the field of vision of the microscope. The presence of TAX (80 µg/mL) in ischemic conditions significantly suppressed the first (reversible) phase of the OGD-induced increase in [Ca^2+^]_i_ and led to the appearance of a long-lag phase before the onset of the second phase of a global increase in [Ca^2+^]_i_ in the neurons (Figure 4A—TAX (in media)) and astrocytes (Figure 4B—TAX (in media)). In this case, necrotic death of cells under OGD in the presence of TAX decreased to 58% (Figure 4C—TAX (in media) + OGD; Figure 4D—TAX (in media) + OGD). The application (nor preincubation) of 80 µg/mL aqTAX to the oxygen- and glucose-deprived medium resulted in the complete suppression of the first phase of the OGD-induced increase in [Ca^2+^]_i_ in the neurons (Figure 4A—aqTAX (in media)) and astrocytes (Figure 4B—aqTAX (in media)), the appearance of a more pronounced lag phase before the global increase in [Ca^2+^]_i_, and a significant inhibition of the global increase in [Ca^2+^]_i_ itself. The necrotic death of cells, in this case, decreased to 28%.

Preliminary incubation of cortical cells with TAX for 40 min suppressed the first phase of the OGD-induced increase in [Ca^2+^]_i_ in the neurons (Figure 4A—TAX (40 min)) and astrocytes (Figure 4B—TAX (40 min)). In this case, the trend towards an increase in the lag phase before the global increase in [Ca^2+^]_i_ was retained; but, in the neurons, the global growth in [Ca^2+^]_i_ itself was substantially inhibited (Figure 4A). However, the preincubation did not eliminate the so-called “irreversible phase” of Ca^2+^ release. After a 40 min incubation of cells with TAX before the induction of ischemia, an even stronger reduction in necrosis occurred (Figure 4C—TAX (40 min) + OGD), and 32% of the cells died, on average (Figure 4D—TAX (40 min) + OGD).

The incubation of cortical cells with aqTAX for 40 min before the development of ischemia completely suppressed the first phase and nearly completely the second phase of the OGD-induced increase in [Ca^2+^]_i_ in the neurons (Figure 4A—aqTAX (40 min)) and astrocytes (Figure 4B—aqTAX (40 min)), which correlated with an almost complete decrease in the amount of PI-positive cells (Figure 4C—aqTAX (40 min) + OGD). After preliminary incubation of the cells with aqTAX under hypoxic conditions, no more than 10% of the cells died (Figure 4D—aqTAX (40 min) + OGD).

Thus, it was shown using equal concentrations of TAX and aqTAX that aqTAX was significantly more effective in inhibiting the OGD-induced increase in [Ca^2+^]_i_ both in neurons and astrocytes. TAX inhibited the “irreversible phase” very little; it only delayed its appearance, whereas aqTAX almost completely eliminated it. Preliminary incubation of cells with aqTAX for 40 min prior to the induction of OGD also led to the almost complete inhibition of the necrotic death of the cortical cells, which was not observed when using TAX.

### 2.5. Comparison of the Structures of Classical TAX and Its Water-Soluble Form

**Figure 5:**
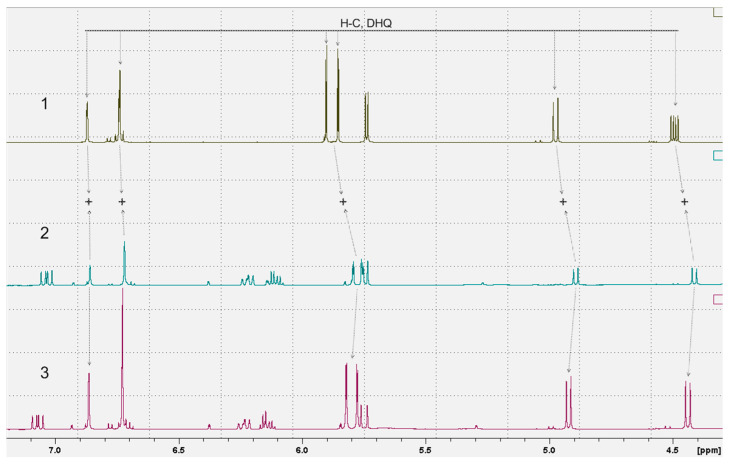
A comparative analysis of ^1^H NMR spectra of TAX and aqTAX. 1. ^1^H NMR spectrum of TAX in DMSO-D6; 2. ^1^H NMR spectrum of AqTAX in DMSO-D6. 3. ^1^H NMR spectrum for a mix of samples 1 and 2.

As mentioned above, the solubility of TAX in water was previously achieved by changing its structure [14,15,16]. To determine whether the state of TAX incorporated in aqTAX was identical to that of classical TAX, we performed a comparative analysis of TAX and aqTAX by NMR (Figure 5).

Figure 5 shows the proton 1D NMR spectra of the samples of TAX, a dietary supplement containing aqTAX, and a mixture of both samples. To determine the possible covalent bond between the TAX and PVP present in the dietary supplement containing aqTAX, a sample of the dietary supplement was mixed with a sample of pure TAX. On the resulting 1H NMR spectrum of the mixture of TAX and aqTAX, the integral values of the proton signals from TAX are equal to the sum of the corresponding signals in the spectra of the samples of pure TAX and the dietary supplement containing aqTAX. This proves the presence of TAX in the composition of the dietary supplement in a molecular form not bound by a covalent bond with other constituents of the dietary supplement. Otherwise, we would have observed TAX signals in double quantity with an integrated intensity corresponding to samples 1 and 2. As this is not the case, the conclusion is unambiguous: TAX molecules in dietary supplements are not covalently bonded to other substances.

In all probability, the binding of TAX with PVP in this sample is accomplished through electrostatic interactions.

## 3. Discussion

A comparative study of the antihypoxic effects of classical TAX and its water-soluble form aqTAX in cultured cerebral cortex cells in ischemia/reoxygenation showed that aqTAX had a much more pronounced protective effect compared with its insoluble form. The compound exhibited antihypoxic activity at significantly lower concentrations.

The study of the effect of both taxifolin forms on cell survival as well as apoptosis and necrosis in cerebral cortex cell cultures under hypoxic conditions showed that aqTAX produces a pronounced effect on cell survival even at a concentration of 10 µg/mL, whereas the concentration of TAX for the manifestation of this effect should be increased almost threefold. As for necrosis that develops in hypoxia, the preincubation of cells with 10 µg/mL AqTAX almost completely inhibits it, whereas a dose of 80 µg/mL is required to attain a similar effect using TAX (Figure 1). The necessity of using high doses of TAX to attain a beneficial effect was confirmed by numerous literature data. Thus, it was proven on some pathological models that the protective effect of TAX against cell death occurs at concentrations of 50–100 μM [9,21,22]. In this case, Wang et al. showed that preincubation with taxifolin at low doses of 10–20 μM did not prevent cell death by the Aβ42 oligomer in SH-SY5Y cells [22].

The effects of TAX on survival and the development of cell necrosis in hypoxia are commonly attributed to its antioxidant activity. The brain is highly vulnerable to oxidative damage due to enhanced oxygen consumption, a high iron content, the presence of excess unsaturated fatty acids, and low activities of detoxifying enzymes [13,23,24].

Cerebral ischemia is known to lead to excessive production of ROS and an increase in their levels, which are regulated by endogenous antioxidant enzymes, including SOD, CAT, and GPX (glutathione peroxidases). As a result, when the activities of these enzymes are insufficient to cope with the elimination of the increased contents of ROS during hypoxia, oxidative stress develops in tissues [25,26].

Presumably, the ability to exhibit antioxidant activity is a necessary but an insufficient property of TAX to explain its neuroprotective action during hypoxia. Also of importance are the ability of this compound to modulate intracellular signaling and transcription factors, to increase the expression of antioxidant proteins and proteins contributing to survival, and to regulate inflammation processes [11].

In our experiments, a preincubation of cortical cells with aqTAX increased the levels of the basic expressions of the genes encoding thioredoxin reductases and glutathione peroxidases, and this trend remained after OGD/R. TAX, in this case, did not affect the basic expressions of these genes (Figure 2 and Figure 3). Thus, aqTAX appeared to be more effective than TAX, which explained its more pronounced cytoprotective effect. It is known that TAX is capable of inhibiting oxidative stress and inflammation through the upregulation of the pAMPK level, along with the activation of the Nrf2/HO-1 signaling pathway [27]. This agreed well with our data, indicating that both taxifolin forms enhanced the basic and the OGD/R-induced expressions of HO-1, with aqTAX being many times more effective.

It is known from the literature that the chronic administration of TAX enhances the activities of redox status proteins, transcription factors, and signaling proteins responsible for cell survival under normal conditions and mild oxidative stress [28]. This agreed well with our data, indicating that a preliminary incubation of cortical cells for 24 h with TAX and aqTAX increased the basic expressions of proteins responsible for cell survival. The effect remained also after the induction of ischemia in the cells. In this case, aqTAX also exhibited a higher activity than TAX.

In ischemic stroke, it is known that oxidative stress activates apoptotic and necrotic signaling pathways, and TAX can act in this process not only as an antioxidant but also as a modulator of transcription factors [29]. Moreover, it is capable of binding to the ATP-specific sites of enzymes and receptors, thereby regulating their activities [30,31].

A comparative analysis of the protective actions of the two taxifolin forms on the Ca^2+^ dynamics in cortical cells in ischemia showed a more pronounced effect of aqTAX on this process. We showed that the presence of TAX under ischemia-like conditions little affected the global increase in [Ca^2+^]_i_ and cell death, and only the preliminary incubation of cells with the compound for 40 min inhibited cell death (Figure 4). AqTAX in the ischemic medium was capable of significantly and more rapidly reducing the global increase in [Ca^2+^]_i_ after OGD, compared with TAX, which was probably due to the high ability of this taxifolin form to more easily penetrate through neurons and bind to the intracellular sites of ionotropic excitatory glutamate receptors, inhibiting their activities. As described in the literature, these effects were achieved only after long-term (for 24 h) incubation with TAX, which was believed to be related to the ability of TAX to increase the expressions of genes regulating the balance between cell survival and cell death [9].

It has been found previously that TAX is capable of accumulating in mitochondria and protecting cells against pathologies associated with mitochondrial dysfunctions [32,33]. It is able to modulate the mitochondrial redox status and the mitochondrial permeability transition pore opening, as well as enhance mitochondrial biogenesis [34,35]. This is probably why the preincubation of cells with TAX and aqTAX for 40 min in our experiments led to their accumulations in the mitochondria of the neurons and astrocytes, which enabled the cells to maintain the activities of the mitochondria for a long time and not to increase ATP production under the conditions of OGD. Apparently, this explained the occurrence of a long-lag phase before the onset of the global OGD-induced increase in [Ca^2+^]_i_, which we recorded after the preincubation of cells with these compounds (Figure 4). At the same time, the ability of aqTAX to more effectively penetrate inside cells enabled it to accumulate in mitochondria at a higher concentration; we observed not only the appearance of a lag phase but also the almost complete suppression of the global increase in [Ca^2+^]_i_ during OGD, which did not occur when using the classical TAX.

The efficiency of the penetration of TAX and, as a consequence, a decrease in its working dose were achieved as a rule by long-term preincubations or chemical modifications [13]. Recently, a selenium–taxifolin nanocomplex was developed, which possessed high antioxidant properties and exerted antiapoptotic and anti-inflammatory effects; the complex showed a high cytoprotective efficacy in cortical cells in ischemia/reoxygenation and by the action of exogenous H_2_O_2_ [13]. 7-O-Taxifolin esters were synthesized, which exhibited a pronounced neuroprotective effect against oxidative stress and neuroinflammation in tests on the models of HT22 and BV-2 cells. In the same work, on a model of the Aβ25-35-induced memory impairment in AD mice, these compounds improved short-time memory defects [36]. However, in all these cases, the structure of TAX changed, whereas the structure of dihydroquercetin remained unchanged in aqTAX, as indicated by our NMR study (Figure 5). Dihydroquercetin, in this case, was bound with PVP, which endowed it with water solubility, evidently, by electrostatic interactions.

In models of Huntington’s disease, TAX attenuates motor coordination deficits and anxiety, decreases the proliferations of microglia, and increases the number of astrocytes [37]. In our experiments, aqTAX produced a more pronounced effect on the Ca^2+^ signaling system of astrocytes than the classical form of TAX. Astrocytes play an important role in neuroinflammation by affecting the secretion of some proinflammatory and neurotoxic factors, such as TNF-α, IL-1, IL-6, and NO [38]. TAX, in the concentration range of 25–100 μM, protected glial cells from apoptosis caused by the overproduction of ROS after the application of H_2_O_2_ [39] through the activation of HO-1 and the inhibition of the IL-1β-induced release of IL-6 and IL-8 [40]. The activation of HO-1 after the application of TAX led to a decrease in the expression of inducible NO synthase during cerebral inflammation [41]. It was also shown that TAX inhibited the expression of the proinflammatory proteins IL-1β and TNF-α in glial cells in models of inflammation [42]. Our experiments indicated that both TAX forms inhibited the expression of the genes of proapoptotic proteins Casp-3, p53, Bcl-xL, and Fas with almost equal efficiency, but the preincubation with aqTAX resulted in a more distinct expression of antiapoptotic Bcl-2. The inhibition of apoptosis by these compounds probably occurred through the inhibition of PUMA, which regulated the expression of these proteins, as was shown earlier for TAX [43,44]. Thus, both taxifolin forms produced marked anti-inflammatory and antiapoptotic effects; however, the efficiency of aqTAX was significantly higher than that of TAX.

A number of studies on the activity of TAX in humans under various physiological conditions confirmed its nontoxicity along with its excellent biological activity. This made it possible to use the compound in the form of a dietary additive as an antioxidant, an immunostimulatory agent, and a cardioprotector for athletes [11]. It was shown that the concentration of TAX in the nervous tissue was maintained for 24 h, whereas its intracellular level was maintained over a longer time in the epithelium [28,45,46,47]. Therefore, an optimal condition providing a chronic action on a monolayer culture of the cerebral cortex was preliminary incubation for 24 h.

It is known that the applicability of dihydroquercetin (TAX) and quercetin is significantly limited by the fact that their cytotoxic and proapoptotic effects manifest themselves only at high concentrations (more than 100 µM in a cell culture) and are accomplished through the autoxidation and generation of toxic quinones. It was shown that their application is restricted by poor bioavailability, which results from low water solubility and poor permeability of the blood–brain barrier [48,49]. It was also found that the abuse of TAX can lead to side effects in the form of headaches, tingling in the stomach, and others [50]. Based on the comparative data obtained in this work, we believe that aqTAX, which is active at significantly lower concentrations and shows a higher bioaccessibility, is a more effective and, therefore, more promising agent for the treatment of diseases associated with the development of oxidative stress and mitochondrial dysfunction. The study of the mechanism of its action on the body in response to various stress factors is of great interest for modern biology.

## 4. Materials and Methods

Experimental protocols were approved by the Bioethics Committee of the Institute of Cell Biophysics (Russia Academy of Sciences). Experiments were carried out according to Act708n (23 August 2010) of the Russian Federation National Ministry of Public Health, which states the rules for the care and use of laboratory animals, and the Council Directive 2010/63 EU of the European Parliament on the protection of animals used for scientific purposes.

### 4.1. Preparation of Mixed Neuroglial Cell Cultures

BALB/c mice were used to obtain cell cultures. Mice were kept in cages 40 × 25 × 15 cm under standard laboratory conditions: a 12 h light circuit, 22 °C. Animals had free access to food and water. For housing, individually ventilated GM500 cages manufactured by Tecniplast (Buguggiate (VA), Italy) with a floor area of 501 cm^2^ were used. In the nests, the harem type of housing (2 females + 1 male) was carried out. Cell cultures from the cerebral cortex were prepared as described in detail previously [51]. Briefly, 0–1-day-old pups were euthanized and decapitated. The extracted tissue was washed with Mg^2+^- and Ca^2+^-free Versene (BioloT, Saint Petersburg, Russia, Cas#1.2.3.2) solution and minced with scissors. Then, tissue fragments were digested with a 1% trypsin (BioloT, Saint Petersburg, Russia, Cas#1.2.2.6) solution for 10 min at 37 °C and washed two times with cold Neurobasal-A medium. A trypsinized tissue was gently pipetted, and the debris was then carefully removed with a pipette tip. The cell suspension obtained was seeded on polyethyleneimine-coated glass coverslips and grown for 10 days in vitro in a culture medium composed of Neurobasal-A medium (Thermo Fisher Scientific, Carlsbad, CA, USA, Cas#10888022), supplement B-27 (2%, Thermo Fisher Scientific, USA, Cas#17504044), and 0.5 mM GlutaMAX (Thermo Fisher Scientific, USA, Cas#35050061).

The drugs were added into culture medium under sterile conditions in the case of experiments with 24 h preincubation with compounds. Then, the cell cultures were washed with Hank’s balanced salt solution and used in experiments.

### 4.2. Fluorescent Ca^2+^ Measurements

To detect changes in [Ca^2+^]_i_, cell cultures were loaded with Fura-2 (4 µM, 40 min incubation, 37 °C, Thermo Fisher Scientific, USA, F1221). The cells were stained with a probe dissolved in Hank’s balanced salt solution (HBSS, Thermo Fisher Scientific, USA, Cas#88284) composed of (mM): 156 NaCl, 3 KCl, 2 MgSO_4_, 1.25 KH_2_PO_4_, 2 CaCl_2_, 10 glucose, and 10 HEPES, pH 7.4. To measure [Ca^2+^]_i_, a system based on a motorized inverted microscope Leica DMI6000B (Leica, Wetzlar, Germany) with a high-speed monochrome CCD-camera HAMAMATSU C9100 was used. For the excitation and registration of Fura-2 fluorescence, a FU-2 filter set (Leica, Germany) with excitation filters BP340/30 and BP387/15, a beam splitter FT-410, and an emission filter BP510/84 were used. An illuminator Leica EL6000 with a high-pressure mercury lamp served as a source of excitation light. Neurons and astrocytes were distinguished by short-term applications of 35 mM KCl before the main experiments, as described previously [52]. Briefly, KCl induced the depolarization of excitable cells, which contained a wide range of voltage-gated cation channels. KCl-induced depolarization promoted the opening of voltage-gated calcium channels in neurons (predominantly L-type channels). The conductivities and densities of cation channels in astrocytes were insufficient to evoke high-amplitude Ca^2+^ responses to KCl applications. All the Ca^2+^ signals were presented as a 340/380 ratio of Fura-2 fluorescence.

### 4.3. A Technique for Simulation of Ischemia-like Conditions

Ischemia-like conditions (oxygen–glucose deprivation, OGD) were created by omitting glucose (HBSS medium without glucose) and by replacing dissolved oxygen with argon in a leak-proof system [53]. The level of oxygen in the medium was measured using a Clark electrode. Oxygen tension reached the values of 30–40 mm Hg or less within 20 min after the beginning of displacement. Ischemia-like conditions lasting for 40 min were created by delivering the oxygen–glucose-deprived medium into a chamber with cultured cortical cells. To prevent the contact of the OGD medium with the atmospheric air, a constant argon flow was fed into the experimental chamber.

### 4.4. Assessment of Cell Viability and Apoptosis

The number of dead cells in cell cultures before and after OGD was evaluated by propidium iodide (PI, Merck, Rahway, NJ, USA, Cas#25535-16-4) (1 µM). Cells were stained for 5 min with probes diluted in HBSS and then rinsed with HBSS. Fluorescence of the probes was recorded by means of an inverted fluorescent microscope Zeiss Axio Observer Z1 (Carl Zeiss Microscopy GmbH, Jena, Germany) using Filter Set 20. Cell death induced by OGD was assessed by PI staining (1 µM) before and after the exposures in the same microscopic field. As PI stained both dead astrocytes and neurons, the types of cells were identified by analyzing calcium signals after the application of 35 mM KCl before OGD. Neurons were identified by a fast transient calcium signal upon KCl application, as described previously. Furthermore, Ca^2+^ signals (presence or absence of a global increase in [Ca^2+^]_i_ during OGD) served as additional indicators of cell viability [18].

The number of dead cells in cell cultures before and after 40 min OGD and 24 h reoxygenation was estimated by Hoechst 33342 (2 µM, Thermo Fisher Scientific, USA, #62249) and PI (1 µM). Cells were stained for 5 min with probes diluted in HBSS and then rinsed with HBSS. The fluorescence of the probes was detected with an inverted fluorescent microscope Zeiss AxioObserver Z1 using Filter Set 01 and Filter Set 20. The discrimination of early and late apoptotic cells was performed as described previously [54]. Five different areas of each cell culture were analyzed. Each experimental group consisted of three cell cultures from different passages.

### 4.5. Extraction of RNA

To isolate total RNA [55], we used neuroglial cell cultures obtained from the cerebral cortexes of mice cultivated in a CO_2_ incubator for up to 10 days. Isolation of total RNA from cells was performed using a total RNA isolation reagent, the ExtractRNA reagent (Evrogen JSC, Moscow, Russia, #BC032), containing a solution of phenol and guanidine isothiocyanate. The reagent was added to a Petri dish with a cell monolayer at the rate of 1 mL per 10 cm2 of the surface, after which total RNA was isolated according to the manufacturer’s protocol. The quality of RNA isolation was checked by electrophoresis in 1% agarose gel, as well as on a spectrophotometer at a wavelength of 260 nm and by calculating the ratio of absorption values 260/280 and 260/230. To prevent contamination of genomic DNA RNA samples, they were treated with DNase I (Thermo Fisher Scientific, USA, #EN0521) at 37 °C for 1 h, after which the enzyme was inactivated by adding 50 mM EDTA (Thermo Fisher Scientific, USA, #R1021) to the mixture followed by heating to 60 °C for 10 min. The concentration of extracted RNA was determined with a NanoDrop 1000c spectrophotometer.

The reverse transcription reaction was carried out according to the protocol, using a kit of reagents for the synthesis of the first strand of the cDNA (Evrogen, Russia, #SK021) containing the reverse transcriptase of mouse leukemia virus (MMLV). The content of total RNA in the reaction mixture was 2 μg; the reaction was carried out in the presence of oligo (dT) primers.

### 4.6. Quantitative Real-Time Polymerase Chain Reaction (RT-qPCR)

Each PCR was performed in a mixture (25 μL) composed of 5 μL of qPCRmix-HS SYBR (Evrogen, Moscow, Russia, #PK147L), 1 μL (0.2 μM) of the primer solution, 17 μL of water (RNase-free), and 1 μL of cDNA. A Dtlite Real-Time PCR System (DNA-Technology, Moscow, Russia) was used for amplification. The sequences of the used primers are presented in Table 1. All the sequences were designed based on the analysis of the nucleotide sequences of the existing gene isoforms and were specific for the mice with FAST PCR 5.4 and NCBI Primer-BLAST software (https://www.ncbi.nlm.nih.gov/tools/primer-blast/index.cgi?GROUP_TARGET=on, accessed on 18 May 2023). The amplification process consisted of the initial 5 min denaturation at 95 °C, 40 cycles of 30 s denaturation at 95 °C, 20 s annealing at 60–62 °C, and a 20 s extension step at 72 °C. The final extension was performed for 10 min at 72 °C. All the sequences were designed using FAST PCR 5.4 and NCBI Primer-BLAST software. The data were analyzed with the Dtlite software (https://dna-technology.com/software (accessed on 18 May 2023), DNA-Technology, Moscow, Russia). The expressions of the genes were normalized to the gene encoding glyceraldehyde 3-phosphate dehydrogenase (GAPDH) [55]. The changes in the levels of mRNA expressions of the studied genes, before and after treatment, were determined by the formula 2^–ΔΔCt^, where ΔΔCt was the difference in ΔCt values for each gene before and after cell treatment. Each cycle of the experiment was repeated three or more times. The data were analyzed using the Livak’s method [56].

### 4.7. Study of the State of TAX in AqTAX by NMR Spectroscopy

Three samples containing DHQ were tested: TAX, aqTAX, and a mixture of TAX and aqTAX. TAX and aqTAX samples were in a lyophilized form. Each sample was sequentially dissolved in a deuterated solvent: 1—DMSO-D6 (600 μL) was added to a weighed sample of dry TAX; 2—DMSO-D6 (600 μL) was added to a weighed sample of a dry aqTAX sample; 3—samples 1 and 2, after registration of their NMR spectra, were mixed in equal proportions. Samples 600 μL in volume were placed in NMR ampules Ø 5 mm.

One-dimensional (1D) and two-dimensional (2D) NMR spectra were recorded on a Bruker 600 AVANCE III spectrometer (Bruker BioSpin, Rheinstetten, Germany). All measurements were performed at 298 K. Standard pulse sequences from the Bruker pulse sequence library were used in experiments. One-dimensional proton spectra were recorded using the simplest 1D pulse sequence ZG. The working frequency for protons was 600 MHz, the free induction decay (FID) was recorded for aq = 3.42 s at 96k points, and the spectrum width was 24 ppm, with a 90° pulse of 10 μs. The time interval between scans during the recording of NMR spectra was 10 s, which was sufficient for the proton relaxations in all the compounds of the samples. Two-dimensional spectra of the homonuclear (1H–1H) spin–spin COSY (sequence COSYGPPRQF) for samples were recorded throughout the region containing signals in the 1D NMR spectra. The delay between COSY pulses was 1 s, and the size of data was of the order of 2048/512 bytes. To attain a reasonable signal/noise ratio, the numbers of the scans were 64 and 128 for the 1D experiments and 2 for the 2D spectra. Chemical shifts were calibrated against the DMSO signal at 2.5 ppm, which served as an internal standard. The processing of spectra and calculation of integrals were performed using the TOPSPIN program (Bruker, Billerica, MA, USA). The state of classical TAX and its soluble form were evaluated by comparing the spectra of the original compounds with the spectrum obtained for a mixture of these two forms. 

### 4.8. Statistical Analysis

The data were obtained for at least three cell cultures from two to three different passages. All values were given as means ± standard error of the means. Statistical analysis was performed by the paired *t*-test. Differences were significant at * *p* < 0.05, ** *p* < 0.01, and *** *p* < 0.001. n/s—data not significant (*p* > 0.05). MS Excel, ImageJ (https://imagej.nih.gov/ij/download.html (accessed on 18 May 2023), Java 1.6.0_12, RRID: SCR_003070, LOCI, University of Wisconsin, Madison, WI, USA), Origin 2016 (OriginLab, Northampton, MA, USA), and Prism GraphPad 7 (GraphPad Software, RRID: SCR_002798) software were used for data and statistical analyses.

## 5. Conclusions

A comparative study of the antihypoxic actions of classical dihydroquercetin (TAX) and its water-soluble form showed that aqTAX produced the effect at significantly lower concentrations compared to TAX. This suggested that significantly lower concentrations of aqTAX would be required than those used commonly in the treatment with TAX in the clinic, which, most likely, would prevent the toxic side effects that occur in the treatment with classical taxifolin.

## Figures and Tables

**Table 1:** Primer sequences for real-time polymerase chain reaction (RT-PCR).

*Gapdh*	Forward 5′-aaggtggtgaagcaggcatc-3′ Reverse 5′-ctcttgctcagtgtccttgc-3′
*Sod1*	Forward 5′-tcgagcagaaggcaagcggtg-3′ Reverse 5′-cggccaatgatggaatgctctcctgag-3′
*Sod2*	Forward 5′-ctcccggcacaagcacagc-3′ Reverse 5′-tcctttgggttctccaccaccctt-3′
*Mao-A*	Forward 5′-ggcggcatctcaggattggct-3′ Reverse 5′-tatgccaaggggttccacacaggt-3′
*Mao-B*	Forward 5′-gcctcagtgtggtggttctggaag-3′ Reverse 5′-cactgggaatctcttggcccatctcatc-3′
*Nos-1*	Forward 5′-gctgcaggtgttcgatgccc-3′ Reverse 5′-ccaaggtagagccatctggctgctt-3′
*Ho-1*	Forward 5′-aggtgatgctgacagaggaac-3′ Reverse5′-tggacagagttcacagcccc-3′
*Cat*	Forward 5′-gctgacacagttcgtgaccctcg-3′ Reverse 5′-acaggcaagtttttgatgccctggt-3′
*TXNRD1*	Forward 5′-caacaaatgttatgcaaaaataatc-3′ Reverse 5′-acactggggcttaacctcag-3′
*TXNRD3*	Forward 5′-ctctttagaaaagtgtgattatatt-3′ Reverse 5′-gcccacatttcattgcagctg-3′
*GPX3*	Forward 5′-gaaaggagatgtgaacgggg-3′ Reverse 5′-gtgggggcatcagttacttc-3′
*GPX4*	Forward 5′-gatgaaagtccagcccaagg-3′ Reverse 5′-gaaggctccaggggtcacag-3′
*Stat3*	Forward 5′-ttctgggcacgaacacaaaagt-3′ Reverse 5′-gcctccattcccacatctctg-3′
*Socs3*	Forward 5′-aagaacctacgcatccagtgtga-3′ Reverse 5′-atgtagtggtgcaccagcttgag-3′
*Casp-3*	Forward 5′-tcagaggcgactactgccggag-3′ Reverse 5′-cgtgagcatggacacaatacacgggt-3′
*Bcl-2*	Forward 5′-ggtgaactgggggaggattg-3′ Reverse 5′-agccaggagaaatcaaacagag-3′
*Bcl-xL*	Forward 5′-tggccacagcagcagtttg-3′ Reverse 5′-tctccggtaccgcagttcaa-3′
*NF-κB*	Forward 5′-ttaaagaaacactcaacagccag-3′ Reverse 5′-ttcagcactcgcacggacac-3′
*RIP1*	Forward 5′-aaggagccctatgagaatgtc-3′ Reverse 5′-acatcctcttctacatattcttc-3′
*TRAIL*	Forward 5′-ctaaccacaacacggaacctg-3′ Reverse 5′-cagcagatggttgatggaggc-3′
*MLKL*	Forward 5′-caaagagcactaaagcagagag-3′ Reverse 5′-ggcaatcctgacccactgg-3′
*TNFα*	Forward 5′-tggaaagacagagggtgcag-3′ Reverse 5′-ttgtcccttgaagagaacctg-3′
*IL-1β*	Forward 5′-aatctcgcagcagcacatcaaca-3′ Reverse 5′-tccacgggaaagacacaggtagc-3′
*IL-10*	Forward 5′-tgtcatcgatttctcccctgtga-3′ Reverse 5′-cattcatggccttgtagacaccttg-3′
*IL-6*	Forward 5′-aaactctaattcatatcttcaac-3′ Reverse 5′-gtccacaaactgatatgcttag-3′
*Gabra1*	Forward 5′-tatctttgggcctggaccctcattctg-3′ Reverse 5′-ccataaggttgtttagccggagcactg-3′
*Gabbr1*	Forward 5′-tcctgtggaagaagaacagggggag-3′ Reverse 5′-cgttggccaggcacttgcg-3′
*Grik1*	Forward 5′-ggaggatgaggcggggacc-3′ Reverse 5′-gcatgctcttcgggaggcttcaaaac-3′
*Grik2*	Forward 5′-ggatgggaaatatggagcccaggatgat-3′ Reverse 5′-tcaggggagagaggattcaggaaggag-3′
*Grin2a*	Forward 5′-gctgacaaggatccgacatccacg-3′ Reverse 5′-gatggaaactctttggggatgagctctgt-3′
*Grin2b*	Forward 5′-ggtgaggtggtcatgaagagggc-3′ Reverse 5′-gggttctgcacaggtacggagttg-3′
*Gria1*	Forward 5′-tgtctacatttatgatgctgaccggggc-3′ Reverse 5′-tcgggagtcacttgtcctccattgc-3′
*Gria2*	Forward 5′-gcatacagataggggggctatttccaagg-3′ Reverse 5′-tgcagtgttgataagcctctgtcactgtc-3′
*p53*	Forward 5′-tgtttaggtcaaggtgtctcc-3′ Reverse 5′-gaacacagcccctaacacag-3′
*fas*	Forward 5′-tggaaaaggagacaggatgacc-3′ Reverse 5′-tttctgctcagctgtgtcttg-3′

## Data Availability

The data presented in this study are available on request from the corresponding author.

## Supplementary Materials

The following supporting information can be downloaded at: https://www.mdpi.com/article/10.3390/ijms241411436/s1.
