# Supplementary material for: A Comparative Analysis of Neuroprotective Properties of Taxifolin and Its Water-Soluble Form in Ischemia of Cerebral Cortical Cells of the Mouse
Source: Int J Mol Sci. 2023 Jul 14;24(14):11436. doi: 10.3390/ijms241411436 (PMC10380368; doi:10.3390/ijms241411436)
Supplement: Supplementary file 1 [file ijms-24-11436-s001.zip › ijms-2433088-supplementary.pdf]

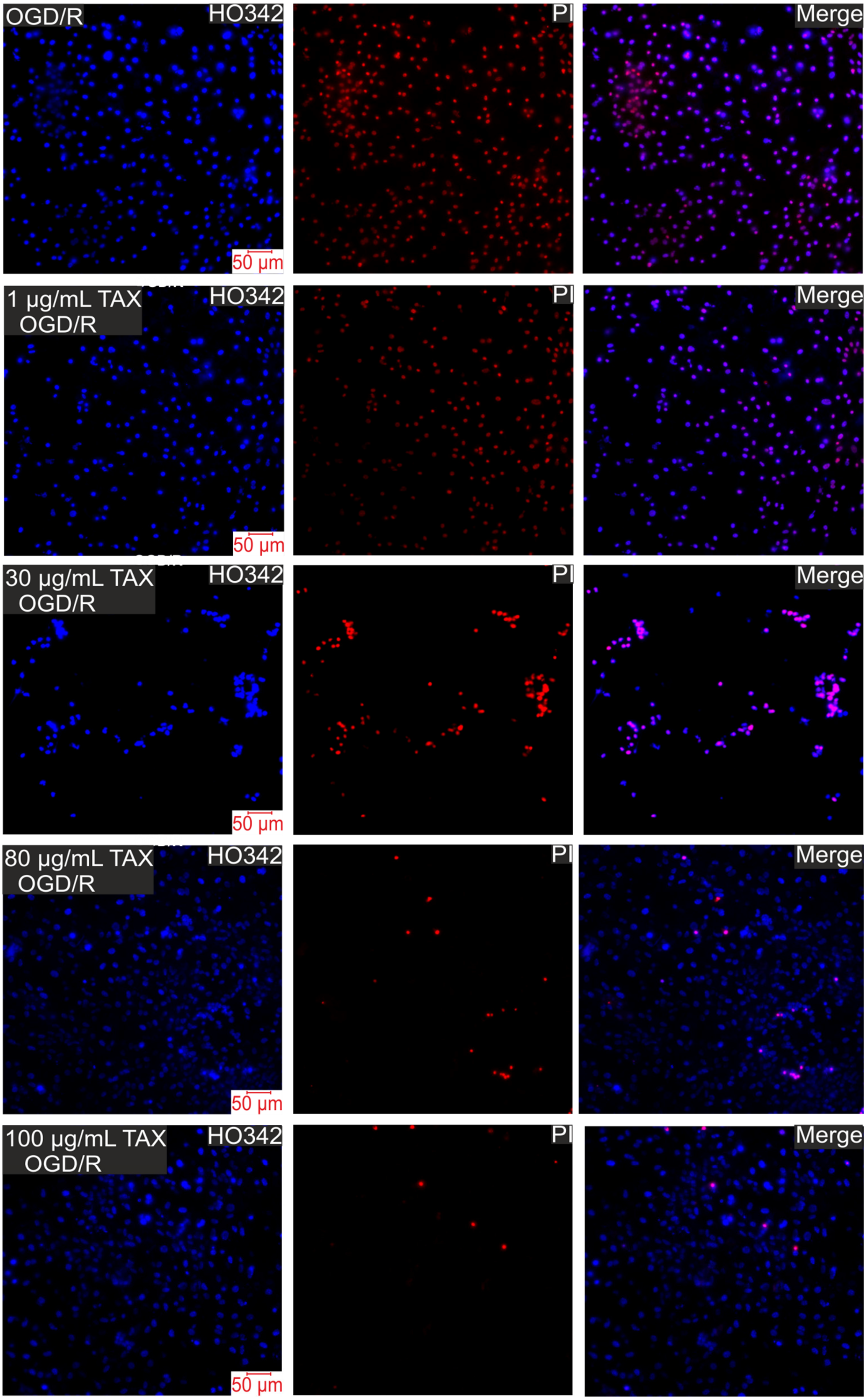
OGD/R
HO342
PI
Merge
50 μm
1 μg/mL TAX
OGD/R
HO342
PI
Merge
50 μm
30 μg/mL TAX
OGD/R
HO342
PI
Merge
50 μm
80 μg/mL TAX
OGD/R
HO342
PI
Merge
50 μm
100 μg/mL TAX
OGD/R
HO342
PI
Merge
50 μm

**SUPPLEMENTARY, FIGURE S1.** The effect of 24-hour incubation of cortical cells with different concentrations of taxifolin (TAX). Double staining of cells with Hoechst 33342 (HO342), Propidium iodide (PI) and merge HO342 with PI. OGD/R – induction of OGD (2 h) and reoxygenation (24 h) without pre-treatment with taxifolin.

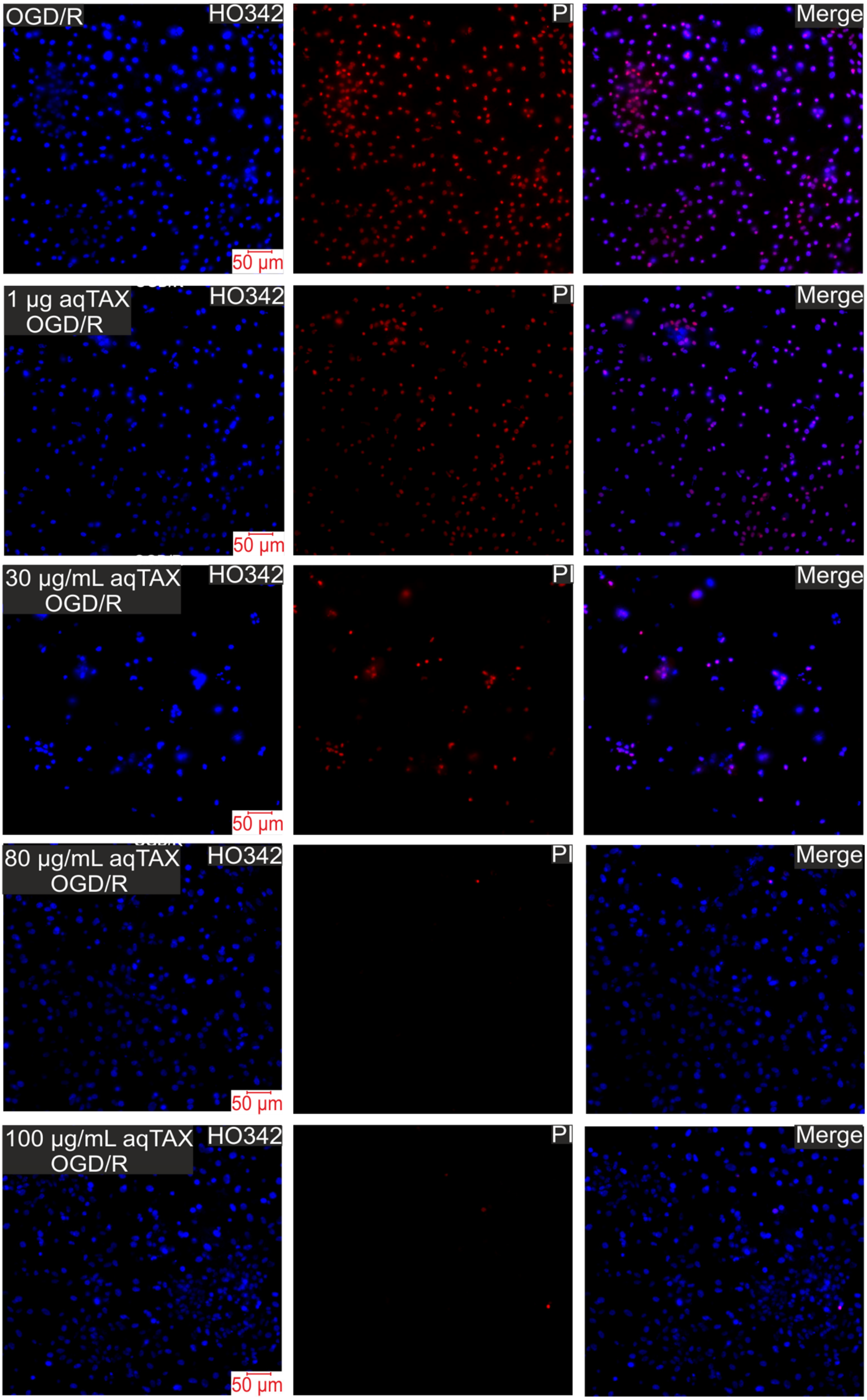
OGD/R
HO342
PI
Merge
50 µm
1 µg aqTAX
OGD/R
30 µg/mL aqTAX
OGD/R
80 µg/mL aqTAX
OGD/R
100 µg/mL aqTAX
OGD/R

**SUPPLEMENTARY, FIGURE S2.** The effect of 24-hour incubation of cortical cells with different concentrations of aqua taxifolin (aqTAX). Double staining of cells with Hoechst 33342 (HO342), Propidium iodide (PI) and merge HO342 with PI. OGD/R – induction of OGD (2 h) and reoxygenation (24 h) without pre-treatment with aqua taxifolin.
